# Predictors of healthcare utilisation among poor older people under the livelihood empowerment against poverty programme in the Atwima Nwabiagya District of Ghana

**DOI:** 10.1186/s12877-020-1473-8

**Published:** 2020-02-27

**Authors:** Williams Agyemang-Duah, Charles Peprah, Francis Arthur-Holmes

**Affiliations:** 1 Department of Planning, Kwame Nkrumah University of Science and Technology, Kumasi, Ghana; 2 Oxford Department of International Development, University of Oxford, Oxford, UK

**Keywords:** Healthcare use, Social and behavioural determinants, Poor older people, Health policy, Ghana

## Abstract

**Background:**

Like many other low- and middle-income countries (LMICs), the Ghanaian healthcare system remains poor which is likely to affect the utilisation of healthcare services, especially among poor older people who are faced with multiple health problems. Yet, factors that explain healthcare use among poor older people in LMICs, particularly Ghana remain largely unexplored. Understanding the predictors of healthcare use among poor older people could have a huge impact on health policies in LMICs including Ghana. This study, therefore, examined factors associated with healthcare use among poor older people under the Livelihood Empowerment Against Poverty (LEAP) programme in the Atwima Nwabiagya District of Ghana.

**Methods:**

Cross-sectional data were obtained from an Ageing, Health, Lifestyle and Health Services (AHLHS) study conducted between 1 and 20 June 2018 (*N* = 200) in Atwima Nwabiagya District, Ghana. Sequential logistic regression models were performed to estimate the variables that predict healthcare use among poor older people. All test results were considered significant at 0.05 or less.

**Results:**

The fully adjusted model showed that respondents aged 85–89 years (AOR = 0.094, CI: 0.007–1.170), acquired basic education (AOR =0.251, CI: 0.085–0.987), received no family support (AOR = 0.771, CI: 0.120–0.620), with no past illness records (AOR = 0.236, CI: 0.057–0.197) and who were not diagnosed of chronic non-communicable diseases (AOR = 0.418, CI: 0.101–0.723) were significantly less likely to utilise health facility compared with their respective counterparts. Moreover, those with no disability (AOR = 19.245, CI: 2.415–29.921) and who consumed low fruits (AOR = 1.435 = CI: 0.552–8.740) and vegetables (AOR = 1.202 = CI: 0.362–10.20) had a higher likelihood to use healthcare.

**Conclusion:**

The study has outlined multiple factors influencing utilisation of healthcare among poor older people under the LEAP programme in Ghana. The results, therefore, validate the importance of social and behavioural determinants of healthcare use in the Ghanaian poor older population. We highlight the need for health planners and stakeholders to consider demographic, socio-economic, health-related and lifestyle factors when formulating health policy for poor older people in Ghana.

## Background

Ghana like any other low-and middle-income countries (LMICs) faces population ageing challenges. Ghanaian older population has increased more than seven-fold from 213,472 in 1960 to 1,643,381 in 2010 [1], which is increasing at a faster pace in the sub-Saharan Africa region. However, most older people are trapped in vicious cycles of poverty in Ghana. The prevalence of poverty among older persons is likely to have a negative effect on their healthcare use patterns. To reduce poverty situation among the older people and to improve their economic conditions and health in Ghana, social policies and programmes, such as the National Social Protection Strategy, National Ageing Policy [2] and the Livelihood Empowerment Against Poverty (LEAP) programme, have been introduced.

The LEAP programme is a financial transfer policy funded by the World Bank, the United Nations International Children’s Emergency Fund **(**UNICEF) and the Government of Ghana. It gives financial protection to people and households that are considered extremely poor [3, 4]. Those who benefit from the LEAP programme comprise older people aged 65 years or more, orphaned and vulnerable children, and persons with disabilities [3, 5]. They get a bi-monthly amount of GH¢ 64 and 106 (US$13.42–22.23)^1^ as a minimum and maximum amounts, respectively [6]. It also provides support for older persons to utilise healthcare services [7].

Studies have shown that with increasing ageing, healthcare utilisation by older people is expected to increase [8, 9]. This is because ageing comes with increased morbidities [10] in a form of functional limitations, cognitive declines and psychological distress [11, 12], which partly result from a weak immune system [13]. In Irbid Governorate of Jordan, Alkhawaldeh et al. [14] found that approximately three in four older people utilised medical care services. Also, in Nigeria, Atchessi et al. [15] discovered that 53% of older people utilised healthcare services. In a cross-sectional survey by Awoke et al. [9] in Ghana, 17.8 and 51.7% of older persons were found to utilise private and public healthcare facilities respectively. According to Dei & San Sebastian [16], 52.41% of older people in Ghana frequently utilise public healthcare services. This demonstrates that the prevalence of healthcare use among poor older people differs from one country to the other. Even in the same country, differences in healthcare utilisation rate seem to exist across spatial locations. 

In developing economies, the use of healthcare follows the social and behavioural constructs [17, 18]. A person’s utilisation of healthcare service is measured by predisposing factors (social structure, demographic and health beliefs), enabling factors (income, health insurance enrolment, access to healthcare facilities and transportation) and needs factors (perceived health status and health evaluation), which either facilitate or hinder utilisation of healthcare [17, 18]. The social and behavioural constructs correlate utilisation of healthcare facilities to both individual and structural factors [19]. Also, the Health Belief Model (HBM) postulates that susceptibility, severity, benefits, costs, barriers and cue to action are the main constructs that influence people to take an action [20]. However, people have higher odds to use health commendation, such as consulting healthcare providers, if they have the intention to do so [20, 21]. With the use of the social and behavioural constructs and the HBM, we are able to provide a theoretical context for the study and situate our findings within these frameworks. Besides, the models were used to limit the scope of the relevant data obtained from the field by focusing on specific variables and defining them to facilitate better interpretation and analysis of the findings in a particular framework.

Evidence has shown that socio-demographic factors influence healthcare utilisation [22–26]. Studies further show that smoking [14, 27–30], physical activity, alcohol consumption [31–33], and health status [14, 33–40] influence healthcare utilisation. Despite the growing literature on factors predicting healthcare utilisation among older people, there is a paucity of information on the predictors of healthcare use among poor older people, who are enrolled in the LEAP programme in Ghana. Involving poor older persons under the LEAP programme in the Atwima Nwabiagya District of Ghana, this study examined the proximate factors influencing their healthcare utilisation. Understanding the predictors of healthcare utilisation from the perspective of poor older people in Ghana could provide significant direction for health policies and health services development in later life.

## Methods

### Sample and data

The study relied on data from a wider Ageing, Health, Lifestyle and Health Services (AHLHS) study, a cross-sectional study conducted between 1 and 20 June 2018. The AHLHS study was to explore the determinants of healthcare utilisation among poor older Ghanaians under the LEAP programme in the Atwima Nwabiagya District of Ghana. In this study, healthcare use was defined as seeking medical treatment from a health professional at a hospital, clinic and other health centres. Unlike other works [9, 40–44], this study defined an older person as an individual who has attained 65 years or above [45, 46] which was in line with the method used to enroll poor older people in the LEAP programme in Ghana. The LEAP programme helped us to identify poor older people to participate in the study.

In all, 16 communities were randomly selected from Atwima Nwabiagya District, and in these communities, 200 poor older people were selected using cluster and simple random sampling techniques. The sample size was achieved based on a power calculation with alpha 0.05. To ensure that the study participants came from a different socioeconomic background, all geographical areas were fully represented. Atwima Nwabiagya District was grouped into three geographical areas based on north, central and south delineation, where 32, 67 and 101 of the total sample size were respectively allocated based on proportion. The selection of the study participants was based on an exclusion and inclusion criteria approach. Poor older people who were sick as at the time of the survey were excluded from the study. Those who had attained 65 years or more and were enrolled in the LEAP programme in the study area were included in this study.

Data were collected through an interviewer-administered questionnaire from the study participants. The questionnaire was developed in English but it was read in Twi for easy understanding by the respondents and also because most of them were illiterate who could not read or write. Three research assistants from the Department of Geography and Rural Development at the Kwame Nkrumah University of Science and Technology (KNUST), Kumasi, Ghana, were hired and trained to help in the data collection exercise. They were trained for 2 days and the purpose of the study was explained to them. The data collection process was monitored to ensure that the research assistants were doing the right thing. Each administered questionnaire took between 30 and 40 min. The study approval was granted by the Committee on Human Research Publication and Ethics (CHRPE), KNUST School of Medical Sciences and Komfo Anokye Teaching Hospital, Kumasi, Ghana (**CHRPE/AP/311/18**). The purpose of the study was fully explained to the study respondents for their verbal and written informed consent.

### Measures

#### Outcome variable

The outcome variable was healthcare utilisation which was keyed as a dichotomous variable showing ‘no utilisation’ or ‘utilisation’ of healthcare over the past 12 months before the study. This 12 months estimation rate of healthcare utilisation outcome is found to be consistent with earlier studies [13, 35–50].

### Predictor variables

The predictor variables were grouped in four areas: demographic, socio-economic, health-related and health behaviour variables. The demographic variables, such as gender (1 = male, 2 = female), marital status (1 = single, 2 = married), and ethnicity group (1 = Non- Akan, 2 = Akan), were measured as dichotomous variables, age (years) (1 = 65–69, 2 = 70–74, 3 = 75–79, 4 = 80–84, 5 = 85–89, 6 = 90 or above) was measured as ranked variable and religious group (1 = Christianity, 2 = Islam, 3 = African Traditional Religion) was measured as nominal variable. The socio-economic variables, such as education (1 = no formal education, 2 = basic school education, 3 = high school education) was measured as nominal variable, income (GH¢) (1 = 200 or below, 2 = 201–300, 3 = Above 300) was measured as ranked variable whereas enrollment in the National Health Insurance Scheme (NHIS) (1 = Yes, 0 = No) and family support (1 = Yes, 0 = No), were measured as dichotomous variables. Health-related variables, such as past illness records (1 = Yes, 0 = No), disability status (1 = Yes, 0 = No), chronic Non-Communicable Diseases (NCDs) (1 = Yes, 0 = No) and self-related health (1 = Good health status, 2 = Poor health status) were measured as dichotomous variables [51]. Engagement in physical activity (1 = Yes, 0 = No), consumption of alcohol (1 = Yes, 0 = No), consumption of tobacco (1 = Yes, 0 = No), fruits intake (1 = 5 or more serving fruits per day, 2 = less than 5 serving fruits per day) and vegetables intake (1 = 5 or more serving vegetables per day, 2 = less than 5 serving vegetables per day) were measured as dichotomous variables (see Table 1). In line with other studies, adequate fruits and/ or vegetable intake was defined as consuming 5 or more servings per day [52–54].

**Table 1:** Operationalisation and coding of predictor variables

Variable	Operational Definition	Category	Code
Demographic Variables			
Gender [Dichotomous]	Being a male or female	Male	1
		Female	2
Age (years) [Ranked]	Number of years a respondent obtained at the last birthday	65–69	1
		70–74	2
		75–79	3
		80–84	4
		85–89	5
		90 and above	6
Ethnicity group [Nominal]	The ethnic background of respondent	Non- Akan	1
		Akan	2
Religion [Nominal]	Religious affiliation of respondent	Christianity	1
		Islam	2
		African Traditional Religion	3
Marital status [Dichotomous]	Whether or not a respondent is married or single	Married	1
		Single	2
Socio-economic variables			
Education [ranked]	A completed grade of educational level	No formal education	1
		Basic school education	2
		Senior high school education	3
		College/tertiary	4
Monthly income (GH¢) [Ranked]	Income of respondent every month comprising both cash and kind received from all sources within the month	200 or below	1
		201.00–300.00	2
		Above 300	3
Health Insurance [dichotomous]	Whether or not a respondent has enrolled in health insurance before	Yes	1
		No	0
Family support [dichotomous]	Whether or not a respondent receives support from a family member	Yes	1
		No	0
Health-related variables			
Past illness [Dichotomous]	Whether or not a respondent has been ill for the past three months	Yes	1
		No	0
Current health status [dichotomous]	Current rate of a respondent health status	Good Health	1
		Poor Health	2
Chronic non-communicable diseases(NCDs)[Dichotomous]	Whether or not a respondent has been diagnosed of any chronic NCDs	Yes	1
		No	0
Disability status [Dichotomous]	Whether or not a respondent has a disability	Yes	1
		No	0
Health behaviour/lifestyle variables			
Engagement in physical activity [Dichotomous]	Whether or not a respondent has undertaken any physical activity for the past month	Yes	1
		No	0
Consumption of alcohol [Dichotomous]	Whether or not a respondent has consumed alcohol in the last year	Yes	1
		No	0
Consumption of Tobacco [Dichotomous]	Whether or not a respondent has smoked tobacco in the last one year	Yes	1
		No	0
Fruits intake [Dichotomous]	Whether or not a respondent consume the required serving of fruits in a day	Less than 5 servings of fruits per day	1
		5 or more servings of fruits per day	2
Vegetables intake [Dichotomous]	Whether or not a respondent consume the required serving of vegetables in a day	Less than 5 servings of vegetables per day	1
		5 or more servings of vegetables per day	2

### Data analysis 

Data were verified and cautiously checked for inconsistencies. Cross-reference was then done to the original questionnaires to inform corrections and any necessary modifications [51]. The data were entered into a database and analysed statistically using Statistical Package for the Social Sciences (SPSS) software (Version 16.0). Percentages and frequencies were used to describe the background characteristics of the study sample, prevalence of healthcare use, sources of healthcare information, satisfaction with healthcare use and the number of kilometres respondents cover to access healthcare. Furthermore, sequential logistic regression models were used to estimate the variables associated with healthcare use among the study participants. Four different sets of models were developed to estimate the predictors of healthcare use among poor older people. Model 1 comprised demographic variables. Model 2 consisted of socio-economic variables plus all variables in Model 1. Whereas Model 3 comprised all variables in Model 2 plus health-related variables, Model 4 (full Model) consisted of all variables in Model 3 plus health behaviour variables. The test results were considered significant at 0.05 or less.

## Results

### Sample characteristics of the study participants

Table 2 presents data on the socio-demographic characteristics of the study participants. Most of the study participants were females (78%), aged 65–69 (29%), belonged to the Akan ethnic group (84%) and professed Christian faith and doctrine (83%). It was no wonder that most of the study participants were females. This is because about 75% of the beneficiaries who are enrolled in the LEAP programme are females. In addition, 74% were single and 63% had no formal education. The majority of the study participants were enrolled in NHIS (96%) and 75.5% earned monthly income of GH**¢**200 or below. Also, 38% of the study participants received family support.

**Table 2:** Socio-demographic characteristics of the study sample

Variable	Category	n(%)
Gender	Male	44 (22)
	Female	156 (78)
Age (years)	65–69	58 (29)
	70–74	40 (20)
	75–79	23 (11.5)
	80–84	29 (14.5)
	85–89	14 (7)
	90 and above	36 (18)
Ethnic Group	Akan	168 (84)
	Non-Akan	32 (16)
Religion	Christianity	165 (82.5)
	Islam	29 (14.5)
	African Traditional Religion	6 (3)
Marital Status	Single	148 (74)
	Married	52 (26)
Education	No formal education	125 (62.5)
	Basic school education	54 (27)
	High school education	21 (10.5)
Monthly income (GH¢)	200 or below	151 (75.5)
	201.00–300.00	35 (17.5)
	Above 300	14 (7)
Registered for National Health Insurance	Yes	192 (96)
	No	8 (4)
Receive family support	Yes	76 (38)
	No	124 (62)

### Prevalence of healthcare use among the study participants

Table 3 shows the prevalence of healthcare use among the study participants. The results showed that most of the study participants (85%) used healthcare services, covered 2 km to access and utilise healthcare (33%), sourced information on healthcare from family members (54%) and were satisfied with healthcare services utilisation (71%).

**Table 3:** Prevalence healthcare use among the study participants

Variable	Category	n (%)
Use of healthcare in the last one year before the study	Yes	170 (85)
	No	30 (15)
Source of seeking healthcare information	Health professionals	24 (14.1)
	Family	91 (53.5)
	Friends	36 (21.2)
	Media	18 (10.6)
	Drug stores	0 (0)
	Literature	1 (0.6)
Satisfied with healthcare use	Yes	121 (71.2)
	No	49 (28.8)
Kilometres study participants covered to access/use healthcare	Less or equal to 1 km	22 (12.9)
	2 km	56 (32.9)
	3 km	28 (16.5)
	4 Km	11 (6.5)
	5 or more	53 (31.2)

### Predictors of healthcare use among the study participants

A sequential logistic regression analysis was performed to find the factors associated with healthcare use (Table 4). The results in Model 1 indicated that females were 2.694 times more likely to use healthcare compared to their male counterparts (AOR = 2.694, CI: 1.002–7.244). Model 2 showed that uninsured study participants were significantly less likely to use healthcare facilities compared to those who were insured (AOR = 0.096, CI: 0.016–0.585). Furthermore, respondents without family support were significantly less likely to use healthcare compared to their counterparts who received family support (AOR = 0.207; CI: 0.065–0.655). The introduction of socio-economic variables, such as family support and health insurance in Model 2, displaced the significant association between gender and healthcare use. This means that family support and health insurance condensed the relationship between gender and healthcare use. In Model 3, respondents without family support (AOR = 0.125, CI: 0.028–0.552), with no past illness (AOR = 0.088, CI: 0.026–1.292) and reported no chronic NCDs (AOR = 0.159, CI: 0.042–0.602) were significantly less likely to use healthcare compared to their counterparts. The introduction of health-related variables in Model 3 disappeared the relationship between healthcare use and health insurance enrollment entirely insignificant. In Model 4, respondents aged 85–89 years (AOR = 0.094, CI: 0.007–1.170), acquired basic education (AOR =0.251, CI: 0.085–0.987), received no family support (AOR = 0.771, CI: 0.120–0.620), with no past illness records (AOR = 0.236, CI: 0.057–0.197) and who were not diagnosed of chronic NCDs (AOR = 0.418, CI: 0.101–0.723) were significantly less likely to utilise a health facility compared to their respective counterparts. Moreover, those with no disability (AOR = 19.245, CI: 2.415–29.921) and who consumed low fruits (less than five servings per day) (AOR = 1.435 = CI: 0.552–8.740) and vegetables (AOR = 1.202 = CI: 0.362–10.20) were significantly more likely to utilise healthcare facilities.

**Table 4:** Sequential Logistic Regression results on the determinants of healthcare use among poor older people

Variable	Model 1		Model 2		Model 3		Full Model	
	AOR	95% C.I	AOR	95% C.I	AOR	95% C.I	AOR	95% C.I
Demographic								
*Gender* ^*a*^								
Female	2.694*	*(1.002–7.244)*	2.780	(0.969–7.974)	2.386	(0.719–7.920)	2.521	(0.412–12.981)
*Age (years)*								
70–74 ^b^	1.495	(0.404–5.528)	0.912	(0.220–3.776)	0.727	(0.134–3.952)	0.523	(0.127–4.285)
75–79	0.703	(0.182–2.722)	0.549	(0.120–2.511)	0.268	(0.047–1.526)	0.152	(0.017–1.457)
80–84	0.867	(0.244–3.075)	0.438	(0.103–1.860)	0.444	(0.081–2.438)	0.369	(0.030–4.791)
85–89	0.648	(0.140–3.005)	0.419	(0.076–2.308)	0.273	(0.036–2.094)	0.094*	*(0.007–1.170)*
90 and above	1.099	(0.316–3.820)	0.500	(0.121–2.061)	0.419	(0.081–2.17)	0.072	(0.069–2.028)
*Ethnicity*								
Akan ^c^	0.024	(0.001–0.015)	0.013	(0.001–0.017)	0.0021	(0.0025–0.0023)	0.820	(0.184–3.658)
*Religious group*								
Muslim ^d^	0.470	(0.162–1.364)	0.807	(0.238–2.735)	0.437	(0.115–1.659)	1.835	(0.417–8.076)
Traditional	0.355	(0.057–2.225)	0.736	(0.103–5.229)	1.021	(0.064–16.295)	0.801	(0.041–15.797)
*Marital status*								
Married ^e^	2.276	(0. 761–6.810)	2.050	(0.645–6.519)	1.950	(0.559–6.802)	2.450	(0.575–14.266)
Socio-economic								
*Education*								
Basic School ^f^			0.687	(0.252–1.877)	0.387	(0.116–1.292)	0.251*	*(0.085–0.987)*
Senior High School			0.868	(0.199–3.794)	0.442	(0.075–2.588)	0.310	(0.117–2.474)
*Income (GHS)*								
200 or below ^g^			1.343	(0.492–3.661)	1.364	(0.421–4.415)	2.375	(0.548–10.210)
201–300			1.532	(0.396–5.932)	1.744	(0.366–8.309)	3.054	(0.416–23.599)
Above 300			1.641	(0.276–9.742)	1.652	(0.245–11.145)	2.968	(0.509–24.704)
Uninsured			0.096*	*(0.016–0.585)*	0.205	(0.025–1.660)	0.293	(0.015–5.826)
No Family support			0.207*	*(0.065–0.655)*	0.125*	*(0.028–0.552)*	0.771*	*(0.120–0.620)*
Health related								
No Past illness (3 months)					0.088*	*(0.026–1.292)*	0.236*	*(0.057–0.197)*
Poor health status					0.530	(0.179–1.568)	0.822	(0.153–2.943)
No chronic Non-Communicable Diseases(NCDs)					0.159*	*(0.042–0.602)*	0.418*	*(0.101–0.723)*
No Disability					2.303	(0.445–11.925)	19.245*	*(2.415–29.921)*
Health behaviour								
Non- physical activity							1.064	(0.303–3.746)
Non-alcohol users							0.448	(0.133–1.503)
Non-tobacco smokers							0.573	(0.097–3.370)
Inadequate (low) fruits intake (less than 5 serving fruits per day)							1.435^*^	*(0.552–8.740)*
Inadequate (low) vegetable intakes (less than 5 serving vegetables per day)							1.202*	*(0.362–10.20)*
Model fitting information								
-2Log Likelihood		158.617		141.095		110.951		108.696
Hosmer-Lemeshow χ^2^ (significance)		1.387(0.917)		9.986(0.266)		10.620(0.224)		10.879(0.209)
Nagelkerke R^2^		0.089		0.229		0.442		0.457

Italic values indicate significance of *p* value (*p* < 0.05)

Model 1 = Demographic variables; Model 2 = All variables in Model 1 plus socio-economic variable; Model 3 = All variables in Model 2 plus health-related variables; Model 4 = All variables in Model 3 plus health behaviour variable

*CI* Confidence Interval, *OR* Odd Ratio, *AOR* Adjusted Odd Ratio

* *p < 0.05*

^a^ Male is the reference category for gender variable

^b^ 65–69 years is the reference category for age variable

^c^ Non-Akan is the reference category for ethnicity variable

^d^ Christians is the reference category for religious variable

^e^ Single is the reference category for marital status variable

^f^ No formal education is the reference category for education variable

^g^ 200 Ghana cedis or less is the reference category for income variable

## Discussion

This study examined the factors associated with healthcare use among poor older people under the LEAP programme in the Atwima Nwabiagya District of Ghana. As found in this study, 17 in 20 of the respondents reported use of healthcare services in the last 12 months preceding the study. This finding showed a higher utilisation rate of healthcare among the respondents compared to 6 and 40% utilisation rate of mental health services both in the United States [46, 55], 49.6% utilisation rate in Ethiopia [48], 45% reported utilisation rate in rural South Africa [50], 73.8% reported rate in Irbid Governorate of Jordan [14] and 76% in Uganda [56]. The finding is relatively similar to a recent Ghanaian study that reported that 81% of older people utilised formal healthcare services [11]. The higher utilisation of formal healthcare utilisation among the respondents could be attributed to the fact that all the participants were enrolled in the LEAP programme which tries to provide support for poor older people to increase their access to healthcare. Even though the LEAP grant is relatively small to pay for all the medical expenditures of the respondents, it plays a key role in their formal healthcare utilisation.

Moreover, while the prevalence of healthcare use reported in this study appears to be high, other studies in German [33], Korea [37] and rural South African setting [33] have reported a significantly higher utilisation rate of healthcare services of 90, 99 and 96%, respectively. The differences in utilisation rate are subject to disparities in the study design, characteristics of the respondents, study setting, operational definition of healthcare and the period of healthcare use preceding the survey. For instance, whereas some studies [10, 11, 40] defined healthcare utilisation to comprise formal and informal healthcare, this study defined healthcare utilisation in the context of formal healthcare.

In our study, age, education, family support, past illness records, chronic NCDs, disability status, fruits and vegetables intake were significantly associated with healthcare utilisation among the respondents. Age was a predictor of healthcare use among poor older persons. The findings of this study confirm the outcome of previous studies indicating an association between the age of a respondent and healthcare utilisation [9, 48, 57–59]. However, the findings of this study also contradict the findings of other studies [58, 60]. This difference in results might be as a result of how this current study and the previous studies defined older people. Whereas this study defined an older person as a person who is 65 years or above, others [9–11] considered people who have attained 50 years or more as older people. The study found that those between the ages of 85 and 89 years were less likely to utilise healthcare facilities compared to their counterparts who were between 65 and 69 years. Like our study results, Dou et al. [61] also found that inpatient care utilisation reduced with increasing ageing. Although, Awoke et al. [9] reported lower use of healthcare among older people who have attained 80 and above, our finding that respondents aged 85–89 years were less likely to utilise healthcare services is counter-intuitive and appears beyond our expectations. Poor older people aged 85 years or more had a low rate of healthcare use. This is because they have lived beyond the normal survival stage and may tend to care less about health. Besides, most of them are likely to perceive that they can survive without healthcare. Also, the majority of the poor older people aged 85 years or more might have developed dementia and other forms of Alzheimer’s disease which keep their thoughts from use of healthcare services, especially when they do not have any person to take care of them. We strongly admit that the above reasons may not be adequate as there is the possibility of other underlying factors that explain lower odds of utilisation of healthcare services among poor older people who are 85 years or over. Yet, due to the quantitative nature of our study, we were not able to delve into such underlying factors.

Furthermore, the study found a statistically significant association between education and healthcare utilisation among poor older persons. This was in tandem with other empirical studies that have reported an association between education and healthcare use [35, 41]. It is important to note that older persons with primary education have lower odds of using healthcare services compared with those without any educational background [44]. McNamara et al. [39] also reported that in Ireland, people with higher levels of education have fewer visits to a general practitioner (GP) than those with a lower level of education and this, however, conflicts with other studies [22, 62], which could result from differences in the study setting and sample size. These studies suggest that people who are educated are more conscious of their health than the uneducated and thus, they tend to utilise more healthcare services.

Our study also found a statistically significant association between family support and healthcare utilisation among the study participants. It was revealed that study participants who have no family support were less likely to utilise healthcare services compared to those with family support. In the view of Falaha et al. [59], family support during illness acts as a determinant of health service use among older people, and this corroborates our study findings. These findings explain the importance of family support in determining healthcare use among older people. In essence, social support normally received from family members not only provide opportunities for health information delivery but also offer physical and financial assistance for older people in a form of funds for transport and payment of healthcare expenditure as well as providing support for their movement [10]. As pointed by Gyasi et al. [10], older people living with other people are likely to be supported to seek healthcare considering the pressures met at the health facilities and the difficulties involved in travelling.

Again, this study found that past illness influences healthcare utilisation among older people, particularly the poor. Older persons without past illness were less likely to utilise healthcare. This finding supported Andersen & Newman’s [17] framework of healthcare utilisation that persons who experience different past health problems are more likely to demand and make use of medical services. This explained why poor older people with past health problems were having higher odds of using healthcare services in this study. In their framework for the study of access, Aday & Andersen [63] isolated illness level as the most immediate factor that influences people to use healthcare. The finding of this study, therefore, validated the independent frameworks of healthcare utilisation by Andersen & Newman [17] and Aday & Andersen [63]. The higher use of healthcare services among respondents with past illness is as a result of their perceived seriousness of illness and susceptibility as succinctly explained in the HBM. The HBM indicates that people are concerned about whether a disease could lead to death or deteriorate their physical or mental functioning for a long period of time or disable them permanently. As a result, they seek medical care regularly and utilise healthcare services to address their health problems [20, 64, 65].

Poor older persons without chronic NCDs were less likely to utilise healthcare services compared to those with chronic NCDs. In a previous study, older persons with chronic NCDs had significantly higher odds of using healthcare compared to those with acute conditions [36]. Similarly, Kim & Lee [37] reported that people who are suffering from chronic diseases have higher chances of utilising both outpatient and inpatient health services. In line with the finding of Hajek et al. [33], this study reported a significant association between chronic diseases and healthcare utilisation. Interestingly, poor older persons with no disability were significantly more likely to use healthcare facility compared to those with disabilities. This trend is attributed to the various medical and physical accessibility barriers people with disabilities encounter in their quest to use healthcare services which consequently affects their utilisation patterns.

Fruits and vegetables consumption were associated with healthcare utilisation among the study participants. Respondents who consumed low (inadequate) vegetables and fruits in a day were significantly more likely to use healthcare facilities compared to those who consumed high (adequate) fruits and vegetables in a day. This finding is similar to a Ghanaian study that reported that poor older females who had not consumed vegetables in a month were more likely to utilise healthcare services [51]. This may be due to the fact that people who consume adequate fruits and vegetables in a day are likely to perceive their health status as good because of the health benefits associated with fruits and vegetables consumption [66–68]. Thus, whereas adequate consumption of fruits and vegetables improves the health of poor older people, it could also reduce their healthcare expenditure since they would not access healthcare frequently.

This study provides several contributions to policy discussions and research literature concerning healthcae use among poor older people in LMICs. To the best of our knowledge, this is one of the first studies that explore the predictors of healthcare utilisation among poor older people, especially those involved in social protection programmes in Africa. This study provides information to health policy makers to have a better understanding of demographic, socio-economic, health-related and health behaviour correlates of healthcare use among poor older people. Despite the contributions of this study, some limitations are notable. Our findings may be exposed to a potential selective survival bias which may partly be attributed to the selection procedure of the sample and criteria for defining or conceptualising poor older people in this study. Also, given the cross-sectional nature of the study, the direction of the causal relationship between healthcare use and factors influencing healthcare utilisation among poor older people cannot be determined. The results only show a significant association between these variables. Future studies should investigate the causality of the associations and the underlying mechanisms for more detail.

## Conclusion

This study examined factors associated with healthcare utilisation among poor older people under the LEAP programme in the Atwima Nwabiagya District of Ghana. The results revealed that most of the poor older people utilised healthcare services. However, poor older people’s age, education, family support, past illness records, chronic NCDs, disability status, fruits and vegetable intakes were significantly associated with healthcare use. The results, therefore, validate the importance of social and behavioral determinants of healthcare use in the poor older population. We highlight the need for health professionals to intensify their efforts in providing relevant health information on chronic NCDs to poor older people in order to improve their health conditions. The Ghana Health Service, Ministry of Health, and health institutions should organise disease prevention programmes and health promotion activities to reduce the incidence of chronic NCDs among poor older people in Ghana, particularly Atwima Nwabiagya District through good health behaviour and lifestyle, such as engaging in physical activity regularly and consuming fruits and vegetables frequently. Also, health stakeholders should consider demographic, socio-economic, health-related and lifestyle factors in their formulation of health policy for poor older people.

## Data Availability

The datasets used and/or analysed during the current study are available from the corresponding author on reasonable request.

## Abbreviations

AHLHS: Ageing, Health, Lifestyle and Health Services
AOR: Adjusted Odd Ratio
CHRPE: Committee on Human Research and Publication Ethics
CI: Confidence Interval
GP: General Practitioner
HBM: Health Belief Model
LEAP: Livelihood Empowerment against Poverty
LMICs: Low- and Middle-Income Countries
NCDs: Non- Communicable Diseases
NHIS: National Health Insurance Scheme
OR: Odd Ratio
SPSS: Statistical Package for the Social Sciences

## Footnotes

1 Exchange rate as at June 2018 (GH¢ 4.77 = US$1.00)
